# Physiological characterization of maize tolerance to low dose of aluminum, highlighted by promoted leaf growth

**DOI:** 10.1007/s00425-015-2376-3

**Published:** 2015-08-08

**Authors:** Liang Wang, Xian-Wei Fan, Jian-Long Pan, Zhang-Bao Huang, You-Zhi Li

**Affiliations:** State Key Laboratory for Conservation and Utilization of Subtropical Agro-bioresources, Key Laboratory of Ministry of Education for Microbial and Plant Genetic Engineering, College of Life Science and Technology, Guangxi University, 100 Daxue Road, Nanning, 530004 Guangxi People’s Republic of China

**Keywords:** Acidic soils, Aluminum toxicity, Aluminum benefit, Maize, Root

## Abstract

**Effects of a low aluminum (Al) dose were characterized. The Al supplement inhibited root growth but enhanced leaf growth in maize lines with different Al sensitivities.**

High levels of Al are phytotoxic especially in acidic soils. The beneficial effects of low Al levels have been reported in some plant species, but not in maize. Maize is relatively more sensitive to Al toxicity than other cereals. Seedlings, at the three leaf stage, of four Chinese maize foundation parent inbred lines with different Al tolerances, were exposed to complete Hoagland’s nutrient solution at pH 4.5 supplemented with 48 μM Al^3+^ under controlled growth conditions, and then the Al stress (AS) was removed. The leaf and root growth, root cell viability, superoxide dismutase (SOD), peroxidase (POD), catalase (CAT), ions (K^+^, Ca^++^ and Mg^++^), photosynthetic rate and chlorophyll, protein and malondialdehyde contents in tissues were assayed. In conclusion, a low Al dose inhibits root growth but enhances leaf growth in maize. The Al-promoted leaf growth is likely a result of increased protein synthesis, a lowered Ca^++^ level, and the discharge of the growth-inhibitory factors. The Al-promoted leaf growth may be a ‘memory’ effect caused by the earlier AS in maize. Al causes cell wall rupture, and a loss of K^+^, Ca^++^ and Mg^++^ from root cells. CAT is an auxiliary antioxidant enzyme that works selectively with either SOD or POD against AS-related peroxidation, depending on the maize tissue. CAT is a major antioxidant enzyme responsible for root growth, but SOD is important for leaf growth during AS and after its removal. Our results contribute to understanding how low levels of Al affect maize and Al-resistant mechanisms in maize.

## Introduction

Aluminum (Al) is the third most abundant chemical element in the earth’s crust (Pilon-Smits et al. 2009). It is phytotoxic, especially in acidic soils with a pH in the 4.5–5.0 range (Matsumoto 2000). The acidic soils account for ~50 % of the world’s cultivable area (Panda et al. 2009). The phytotoxicity of Al has been studied extensively in organs, tissues, and cells (Kochian 1995; Ma 2000; Sivaguru et al. 2000; Mossor-Pietraszewska 2001; Čiamporová 2002; Ma and Furukawa 2003; Chen 2006; Illéš et al. 2006; Poschenrieder et al. 2008; Giannakoula et al. 2010; Inostroza-Blancheteau et al. 2012). Most studies focused on the inhibition of root growth (Chen 2006; He et al. 2014), and changes in root architecture and elongation under Al stress (AS) (Pilon-Smits et al. 2009). As with other abiotic stresses, AS can result in peroxidation by triggering a greater production of reactive oxygen species (ROS). To detoxify AS-caused ROS, plants employ enzymatic antioxidants, majorly including superoxide dismutases (SOD), peroxidases (POD) and catalases (CAT) (Boscolo et al. 2003; Inostroza-Blancheteau et al. 2012). However, whether these three enzymes equally contribute or selectively cooperate during the detoxification of AS-caused ROS remains unknown.

A few reports have indicated that low or moderate doses of Al have beneficial effects on some plant species, such as *Miscanthus sinensis* (Yoshii 1937), tea plant (Ghanati et al. 2005), and *Melastoma malabathricum* (Watanabe et al. 2005, 2006). Some crops also benefit from Al, such as rice (*Oryza sativa*) (Osaki et al. 1997), triticale (Zhang et al. 1999, 2007) and alfalfa (Zhang et al. 1999, 2007), and soybean (Du et al. 2010).

Maize (*Zea mays* L.) is relatively more sensitive to Al toxicity than other cereals (Doncheva et al. 2005; Poschenrieder et al. 2008). Maize responses to AS have been investigated, but the results have been varied (Boscolo et al. 2003; Giannakoula et al. 2008, 2010; Mihailovic et al. 2008). In soybean, a high level of AS caused plasmolysis, cell wall rupture, and the leakage of cellular contents (Yu et al. 2011). However, apparently no more attention was paid to AS effects on maize cells and their growth. Interestingly, it may be possible to mitigate soil Al toxicity using appropriate methods, such as the application of dolomite (Holmström et al. 2005). This raises the question of whether maize subjected to AS could resume its growth after the removal of AS (RAS).

We characterize the maize responses to AS and RAS at organ, tissue, physiological, and cellular levels in four Chinese maize foundation parent inbred lines (two Al-sensitive lines, and two relatively Al-tolerant lines).

## Materials and methods

### Maize inbred lines and growth conditions

The maize inbred lines used were Huangzao4 (H4), Chang7-2 (C7-2), Ye478 (Y478) and Zheng58 (Z58), of which H4 and C7-2 are more sensitive to AS relative to Y478 and Z58. The maize seeds were kindly supplied by Professor Yu Li of the Institute of Crop Sciences, CAAS. The seeds were surface sterilized by soaking for 12 h at 28 °C in distilled water, and then for 6 min in 75 % ethanol. The surface-sterilized seeds were grown at 28 °C in fresh moist river sand. After emergence, seedlings with the same growth potential were selected and carefully transferred into holes in perforated polystyrene foam boards that were fixed 0.5 cm above the surface of the complete Hoagland’s nutrient solution at pH 7. The nutrient solution was vigorously aerated for 15 min every 1 h, adjusted daily to maintain the pH at 7 ± 0.2, and renewed every 3 days. When reached the three leaf stage, the seedlings were subjected to the AS treatment in the nutrient solution at pH 4.5 supplemented with AlCl_3_·7H_2_O, where the active Al^3+^ concentration was 48 µM.

After a 72-h AS treatment, the seedlings were transferred and grown for 72 h at 28 °C in an Al-free nutrient solution, representing RAS treatment. The seedling control treatment was performed using the Al-free nutrient solution in parallel with the AS treatment. All of the seedlings were grown in a chamber with 60–80 % humidity, a 12 h of light and a constant temperature of 28 °C.

The tissues were sampled at 10 a.m. every 24 h. The sampled tissues were directly used, immediately frozen in liquid nitrogen and then stored at −80 °C, or immediately fixed in a solution containing 5 of 37 % formalin, 90 of 70 % alcohol and 5 of 37 % glacial acetic acid (GAA), depending on the analysis requirements.

### Measurement of leaf and root growth rates

The absolute length of primary roots from the root-stem transition zone to the root tip, and the absolute length of the third leaves from the petiole base to the apex were measured. For both, leaves and roots, growth was expressed as the relative growth rates, which were estimated by (the length under AS/the length under parallel Al-free control) ×100 %.

### Assay of tissue Al ion contents

The Al contents in the tissues were assayed using the conventional S-chromium azure (SCA) chromogenic method. Briefly, the tissues were oven dried. A 0.1-g aliquots of the dried samples was digested for 24 h in 1.5 mL of 2 mM HNO_3_, and then diluted 20 times with deionized water. A 1-mL aliquot of the dilution was transferred to a 25-mL volumetric flask, and then 1 mL HNO_3_ (0.1 M), 2 mL cetyltrimethylammonium bromide (CTAB 5 mM), 2 mL EDTA-Zn (0.05 mM), 2 mL SCA (0.05 %), and 4 mL six-methyl tetramine solution (40 %) were added in that order. Finally, the volume was adjusted to 25 mL volume by adding deionized water, and then sufficiently mixed. The flask was placed for 20 min at room temperature, and the optic density (OD) of the mixed solution at 635 nm was assayed. The resulting OD_635_ values were used to estimate the Al content in the tissues against a standard solution curve prepared with different AlCl_3_·7H_2_O concentrations.

### Preparation of the plant tissue extract

A 0.25-g aliquot of the fresh tissues was homogenized by grinding in 5 mL pre-cooled phosphate buffer solution (PBS) at pH 7.4 containing 1 mM EDTA, and then centrifuged for 20 min at 16,200×*g* at 4 °C. The resulting supernatant was stored at −80 °C.

### Assay of tissue total protein contents

The total protein contents in the tissues were quantified according to the Coomassie Brilliant Blue-based method. In brief, a 1-mL aliquot of the protein extract was fully mixed with 1 mL G-250 Coomassie. Then, the OD_620_ value of the solution was assayed and used to estimate the protein content against the standard solution curve prepared with different calf serum concentrations.

### Assay of antioxidant enzyme activities

The SOD activity was assayed following the methods described in Tang (1999) with some modifications. Briefly, the following solutions were added, in order, to a tuber: 1.5 mL of PBS at pH 7.4, 0.3 mL of 130 mM methionine, 0.3 mL of 750 µM nitroblue tetrazolium (NTB), 0.3 mL of 110 µM EDTA-Na_2_, 0.3 mL of 110 µM riboflavin, 0.1 mL of the plant tissue extract, and 0.5 mL deionized water. The solution mix was allowed to react for 20 min at a light intensity of 3000 lux at 25 °C, and the OD_560_ value of the mix was assayed. A SOD activity unit (U) was defined as a 50 % inhibition of NTB photochemical reduction. The SOD activity was expressed as a specific activity of U mg^−1^ protein.

POD activity was assayed as described in Tang (1999) with minor modifications. Briefly, a 20-µL aliquot of the plant tissue extraction compound was added to, and well mixed with 3 mL PBS (pH 7.4) containing 1 % (v/v) H_2_O_2_ and 5 % (v/v) guaiacol, and then the OD_470_ value was assayed. The POD activity U was defined as an increase in the OD_470_ value of 0.01 min^−1^. The POD activity was determined as the specific activity of ΔA_470_ min^−1^ mg^−1^ protein.

CAT activity was assayed following the methods of Cakmak and Horst (1991) with some modifications. Briefly, a 100-µL aliquot of the plant tissue extract was well mixed with 3 mL PBS (pH 7.4) containing 0.1 M H_2_O_2_, and then its OD_240_ value was assayed. The CAT activity U was defined as a decrease in the OD_240_ value of 0.01 min^−1^.

### Assay of superoxide radicals (SORs)

A 0.5-mL aliquot of the plant tissue extract was well mixed with 0.5 mL PBS (pH 7.4) and 1 mL of 1 mM hydroxylamine hydrochloride, and allowed to react for 1 h at 25 °C. Then, 1 mL sulfanilic acid (17 mM) and 1 mL α-naphthylamine (7 mM) were added, and allowed to react for 20 min at 25 °C. The OD_530_ values of the reaction solution were then measured and used to estimate SOR values against a curve generated by the standard solution, which was made with the above-mentioned reaction solution supplemented with NaNO_2_, at OD_530_. The SOR production was expressed as nM min^−1^ mg^−1^ protein.

### Assay of the malondialdehyde (MDA) content

A 1-mL aliquot of the plant tissue extract was mixed with 2 mL solution containing 0.6 % (m/v) thiobarbituric acid and 10 % (m/v) trichloroacetic acid, reacted for 30 min in a boiling water bath, and immediately cooled on ice. The reaction solution was then centrifuged for 5 min at 11,600 ×*g*. The OD_532_ and OD_450_ values of the resulting supernatant were measured, respectively. The MDA content was estimated using the formula of [(6.45 × OD_532_) − (0.56 × OD_450_)] × plant tissue extraction compound (L)/the sample weight (g).

### Evaluation of root cell viability

The cell viability of fresh roots was evaluated as described previously (Tamás et al. 2006) with some modifications. The roots were rinsed for 5 min with deionized water to fully remove the residues on the surface, and then stained for 30 min in 0.25 % (m/v) Evans blue. After staining, the roots were rinsed for 15 min with deionized water to fully remove the dye on the surface and then photographed.

### Microscopic observation of root tip cells

The root tips (0.5 cm long) from formalin-GAA-alcohol-fixed roots were sectioned lengthwise using a paraffin slicing machine. The thickness of the slices was 6 µm. The resulting slices were stained for 15 min in a solution that was prepared with equal volume of the staining stock solution and 50 % ethanol-GAA solution, where the staining stock solution was composed of 0.66 g hematoxylin, 3 mL GAA, 32 mL glycerol, 32 mL of 95 % ethanol, 1.66 g aluminum potassium sulfate, and 33 mL deionized water. The stained slices were observed by light microscopy.

### Assay of K, Ca, and Mg ions

The tissues were fully oven dried. For each sample, a 0.1-g aliquot of the dried tissues was used to measure K, Ca, and Mg ions in a 6400 atomic absorption spectrophotometer (Shanghai, China) following the conventional atomic absorption analysis method.

### Chlorophyll content assay

A 0.5-g aliquot of fresh leaves, the main veins of which were removed, was homogenized in 10 mL acetone. A 2-mL aliquot of the homogenate was centrifuged for 5 min at 2400 ×*g*. The resulting supernatant was diluted five times with 80 % acetone, and then measured for OD_663_ and OD_645_ values, respectively. The OD values were normalized against the OD value of 80 % acetone, and then used to estimate the chlorophyll content based on the formula: the chlorophyll content [mg g^−1^ fresh weight (FW)] = [(8.02 × OD_663_ + 20.21 × OD_645_) × 10 mL × 5]/(1000 × 0.5).

### Measurement of the photosynthetic rate

The photosynthetic rate measurements were taken at 9:00 a.m. on the middle part of the second leaves by using a Li-6400 portable photosynthesis analyzer (Lincoln, NE, USA) under an artificial red and blue light source.

### Statistical analyses of the data

The significant differences among the data were analyzed through one-way analysis of variance software, and the correlation analyses among the data were conducted based on the Pearson’s correlation coefficient using the SPSS 13.0 software (http://www.spss.com/).

## Results

### Maize growth

**Fig. 1:**
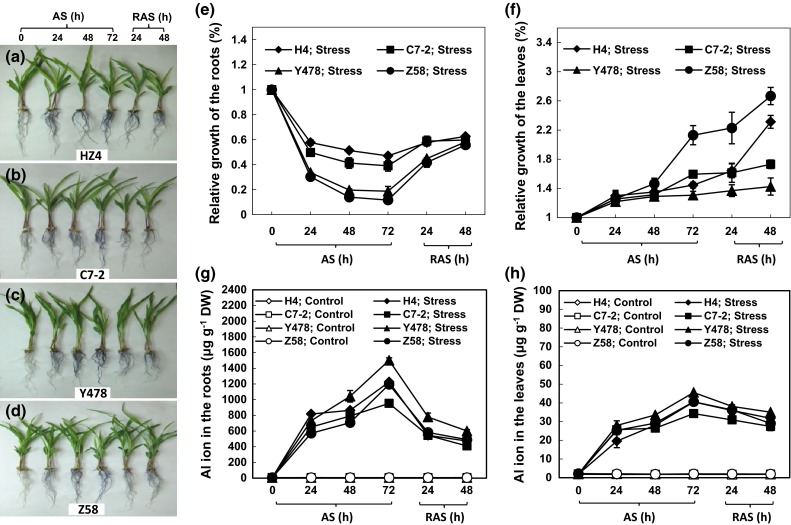
The growth of maize seedlings under AS and after RAS. The phenotypes of the different *maize lines* (**a**–**d**), the relative growth of roots (**e**) and leaves (**f**), and the Al ion contents in roots (**g**) and leaves (**h**). The values are mean ± standard error (SE) from at least five individual seedlings

No Al toxicity-related symptoms were found on the shoots of the tested maize seedlings (Fig. 1a–d). However, the root growth rate was significantly reduced under AS (Fig. 1e). Unexpectedly, the leaf growth rate in all of the maize lines was accelerated under AS, starting at 24 h after AS and increasing more significantly with the duration of AS (Fig. 1f).

After RAS, the roots of the seedlings of the AS-treated maize lines grew in a significantly increased way although the growth rate was still slower than that of the respective control lines (Fig. 1e). Interestingly, the leaves of the AS-treated maize lines had higher growth rates than the respective Al-free controls even after RAS treatment, especially in H4 and Z58 lines (Fig. 1f).

### Al ion content

The Al ion content was much higher in the roots and leaves of the stressed lines under AS than in the respective Al-free controls (Fig. 1g, h). After RAS, the Al content decreased significantly in all of the AS-treated roots (Fig. 1g) and slightly in the stressed leaves (Fig. 1h), when compared with those at the 72-h AS time point.

### SOD activity

**Fig. 2:**
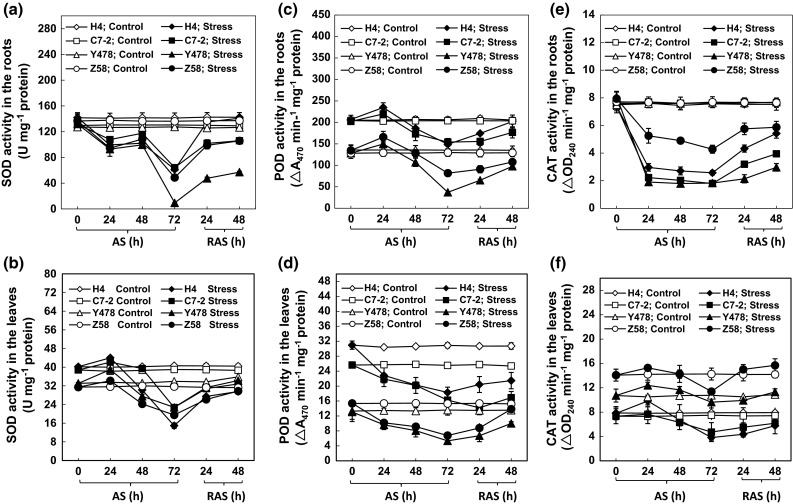
Activities of antioxidant enzymes in the maize tissues under AS and after RAS. The SOD activities in roots (**a**) and leaves (**b**), the POD activities in roots (**c**) and leaves (**d**), and the CAT activities in roots (**e**) and leaves (**f**). The values are mean ± SE from at least five individual seedlings

The SOD activity levels in the roots of the AS-treated maize lines started to significantly decrease 24 h after AS, remained almost unchanged 48 h after AS, and then sharply dropped 72 h after AS, while the activity levels in the roots of the control maize lines did not obviously change (Fig. 2a). The SOD activity levels in the leaves of the AS-treated maize lines were approximate to those in the respective controls for 48 h, but significantly decreased 72 h after AS (Fig. 2b).

The SOD activity levels in the roots of the stressed maize lines at 72 h post AS were obviously increased by RAS (Fig. 2a). However, only the activity in the roots of the AS-treated H4 line reached the level of the corresponding control roots (Fig. 2a). Overall, the SOD activity levels in the leaves of the AS-treated maize lines did recover to levels of respective control leaves after RAS treatment (Fig. 2b).

### Change in POD activity

The POD activity levels in the roots of the AS-treated C7-2 and Y478 lines were almost constant within 48 h after AS but increased slightly in the roots of the AS-treated H4 and Z58 lines at 24 h after AS. However, the activity levels in the roots of the AS-treated maize lines significantly decreased 72 h after AS when compared with the activity levels in respective control roots (Fig. 2c). In contrast, the POD activity levels in the leaves of the AS-treated maize lines started to significantly decrease early at 24 h after AS, and then sluggishly declined with AS when compared with the activity levels in respective control leaves (Fig. 2d).

After 48 h of RAS treatment, the POD activity levels in the roots of the AS-treated maize lines almost reached the activity levels in the respective control roots (Fig. 2c). The POD activity levels in the leaves of AS-treated H4, C7-2 and Y478 lines indeed increased but did not reach the activity levels of the respective control leaves (Fig. 2d). Only the activity level in the leaves of AS-treated Z58 line reached to control level (Fig. 2d).

The decreased activity levels of SOD and POD in the roots and leaves of all of the AS-treated maize lines (Fig. 2a–d) were not in agreement with the results previously reported in maize under AS (Boscolo et al. 2003).

### Change in CAT activity

The CAT activity levels in the roots of the AS-treated maize lines started to significantly decrease 24 h after AS, and then remained almost unchanged during further AS treatment (Fig. 2e). After RAS, the activity levels significantly increased but did not reach the activity levels in the respective control roots (Fig. 2e). During AS and RAS, the changes in the CAT activity levels in the leaves of the AS-treated maize lines (Fig. 2f) were similar to the changes in SOD activity levels in the leaves of the AS-treated maize lines (Fig. 2b).

### MDA content and SOR production

**Fig. 3:**
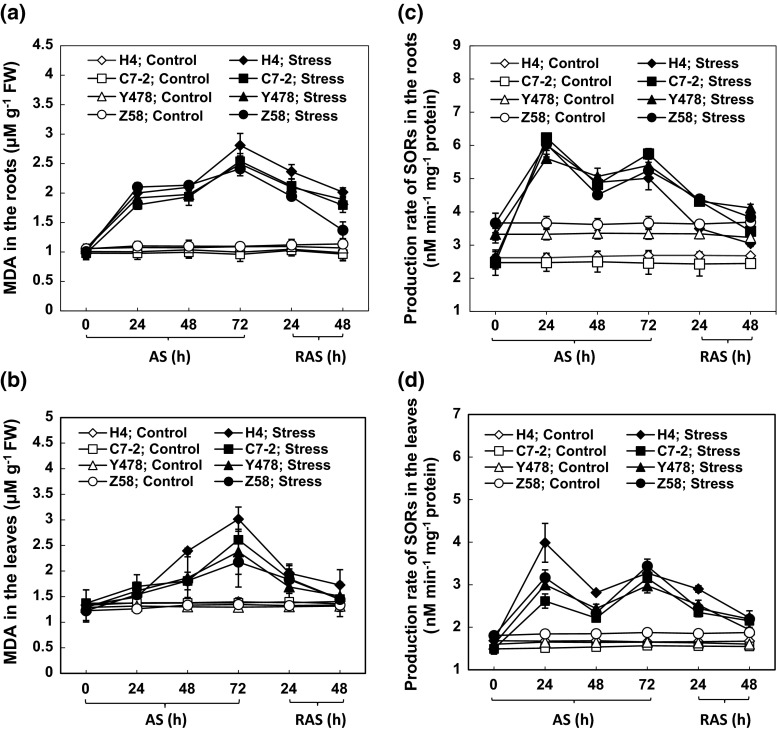
The MAD contents, and SOR production rate in the maize tissues under AS and after RAS. The MDA contents in roots (**a**) and leaves (**b**). The SOR production rates in roots (**c**) and leaves (**d**). The values are mean ± SE from at least five individual seedlings

The MAD contents in the roots of the AS-treated maize lines started to significantly increase 24 h after AS (Fig. 3a). After the RAS treatment, the contents in the roots of the AS-treated maize lines significantly declined when compared with those of the respective roots at the 72-h time point of AS. However, only the MDA content in the roots of the AS-treated Z58 line was similar to that in the corresponding control after RAS treatment (Fig. 3a).

The MDA contents in the leaves of the AS-treated maize lines started to significantly increase 48 h after AS (Fig. 3b), lagging behind the changes in the MDA contents in the roots of the AS-treated maize lines (Fig. 3a). However, the MDA contents in the leaves of the 72-h-stressed maize lines almost recovered after RAS treatment to the respective control level (Fig. 3b).

The increased MDA contents in the roots and leaves of the AS-treated maize lines did not support the previous conclusion that the presence of Al did not cause lipid peroxidation (Boscolo et al. 2003).

With the increase in MDA contents, SOR production in the roots and leaves of all the AS-treated maize lines increased at 24 h, decreased at 48 h, and then increased again at 72 h after AS (Fig. 3c, d).

### Viability of the root cells

**Fig. 4:**
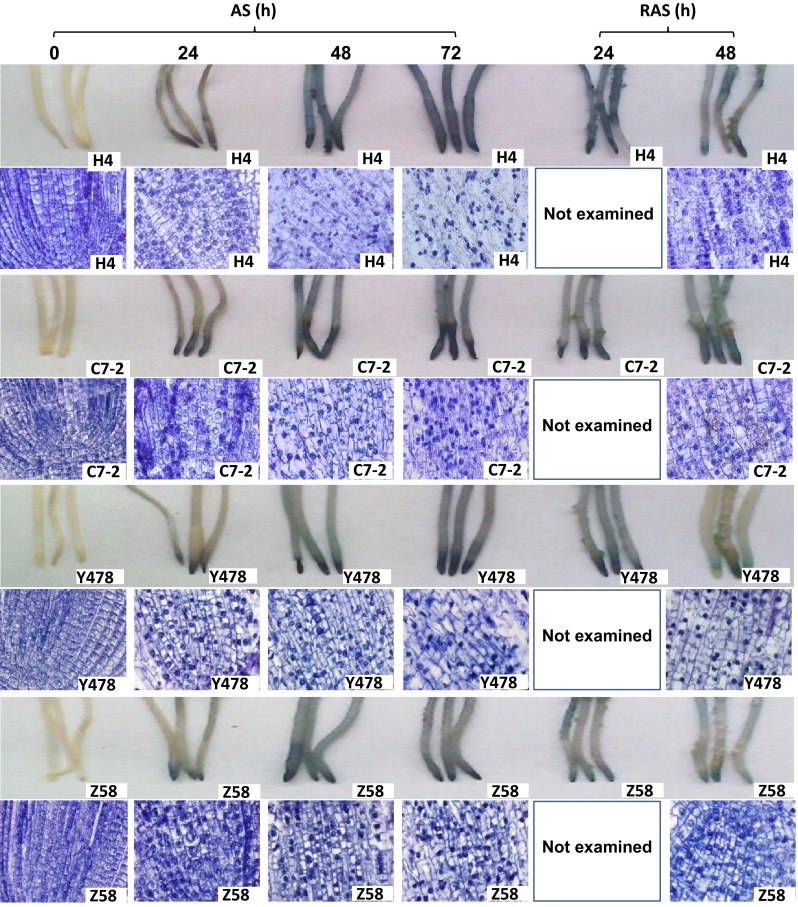
Viability and structure of the fresh root cells under AS and after RAS. The root cell viability was assayed by using Evans blue staining method. For observation of cell structure, the tissues of the root tip zone were sectioned lengthwise by using a paraffin slicing machine and then photographed by light microscopy. The detailed procedures were indicated in “Materials and methods”

The Evans blue staining indicated that decreased root cell viability under AS occurred in cells near the root tips at 24 h after AS, and then was found in the cells in the upper tissues with AS, being more obvious in the roots of the AS-treated H4 and C7-2 lines (Fig. 4). Notably, a decreased root cell viability in Z58 line during AS seemed to be limited to the cells near the root tip zone (Fig. 4). The decrease in root cell viability in the AS-treated maize lines could be alleviated by RAS treatment, especially in the roots of AS-treated Y478 and Z58 lines (Fig. 4).

The cells in the root tip zones of the AS-treated maize lines showed plasmolysis and cell wall rupture, and had concentrated and enlarged nuclei, while the cellular contents leaked (Fig. 4). These symptoms started 48 h after AS, and were more serious in H4, C7-2 and Y478 lines than in Z58 line (Fig. 4). Interestingly, the symptoms were greatly alleviated by 48 h of RAS treatment (Fig. 4).

### K, Ca, and Mg ions in the tissues

**Fig. 5:**
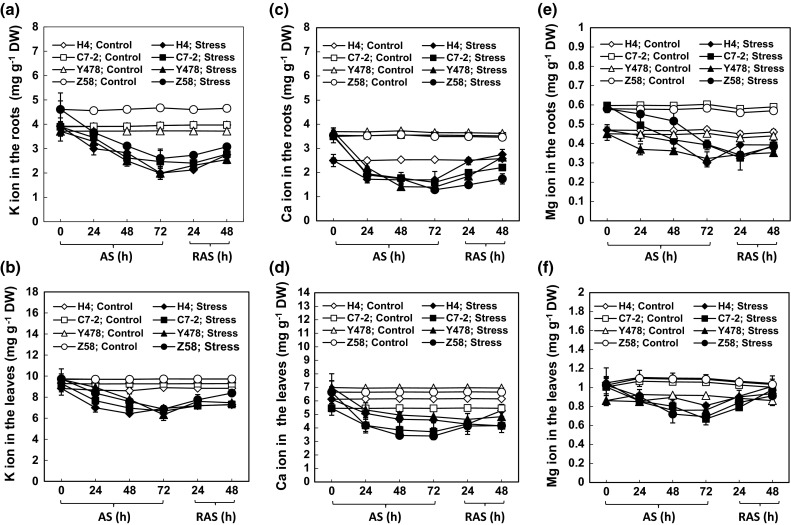
The contents of K (**a**, **b**), Ca (**c**, **d**) and Mg ions (**e**, **f**) in maize roots (**a**, **c**, **e**) and leaves (**b**, **d**, **f**) under AS and after RAS. The values are mean ± SE from at least five individual seedlings

The K, Ca, and Mg ion contents in the roots and leaves of all the AS-treated maize lines declined when compared with the levels in the respective controls (Fig. 5a–f). The contents of these ions in tissues of the AS-treated maize lines were significantly enhanced by RAS treatment (Fig. 5a–f). These results echoed the changes in cell structure in the root tip zone (Fig. 4).

### Protein and chlorophyll contents, and the photosynthetic rate

**Fig. 6:**
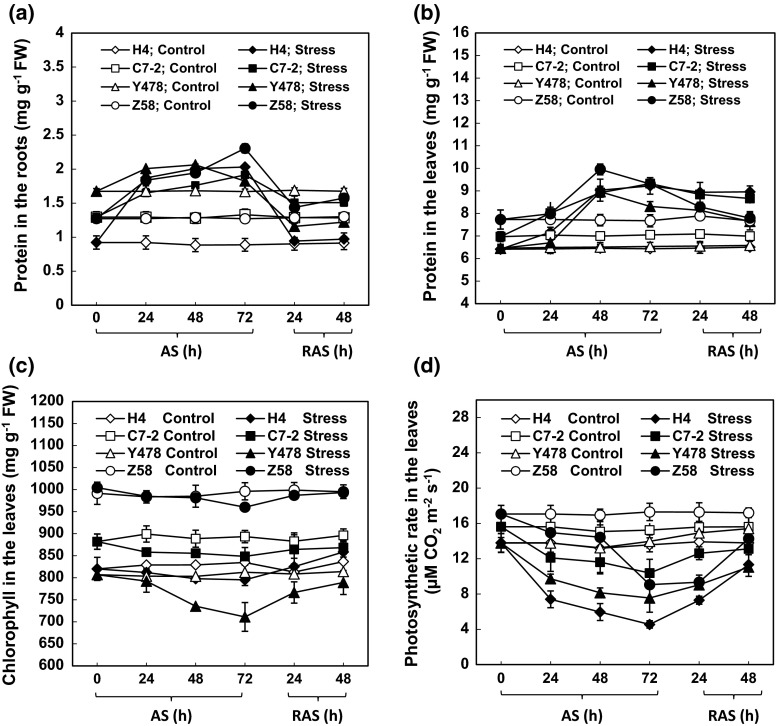
The protein content in maize roots (**a**) and leaves (**b**), and chlorophyll contents (**c**) and photosynthetic rate (**d**) in maize leaves under AS and after RAS. The values are mean ± SE from at least five individual seedlings

The total protein contents were significantly higher in the roots and leaves of the AS-treated maize lines than in the roots and leaves of respective control lines (Fig. 6a, b). The protein content started to significantly increase 24 h after AS in the roots (Fig. 6a) and 48 h after AS in the leaves (Fig. 6b). After RAS treatment, the protein contents in the tissues of the AS-treated maize lines significantly declined when compared with protein content levels in corresponding AS-treated maize lines before RAS (Fig. 6a, b).

The chlorophyll contents in the leaves of the AS-treated Z58 line decreased slightly within 48 h of AS and significantly 72 h after AS when compared with the chlorophyll levels in the control line (Fig. 6c). A significant decrease in the chlorophyll content was found in three AS-treated maize lines (H4, C7-2 and Y478), starting 24 h or 48 h after AS depending on the lines. After RAS treatment, the chlorophyll contents in the leaves of the AS-treated maize lines reached the levels in the respective controls (Fig. 6c).

The photosynthetic rates in the leaves of all the AS-treated maize lines started to significantly decrease 24 h after AS (Fig. 6d). After RAS treatment, the photosynthetic rates in the leaves of the AS-treated maize lines obviously increased when compared with the photosynthetic rate levels in respective maize lines treated by AS for 72 h (Fig. 6d).

## Discussion

Al toxicity in plants occurs in acidic soils (Matsumoto 2000). However, the beneficial effects of low Al doses on plants in acidic soils may occur in both Al-tolerant plants and many Al-stimulated plants (Osaki et al. 1997), and is characterized by growth promotion. The seedlings of Al-tolerant triticale and alfalfa showed large root regrowth during AS (Zhang et al. 1999, 2007). Additionally, lower Al concentrations significantly stimulated the root growth of Al-tolerant soybean PI 416937 (Du et al. 2010).

All of the maize lines tested in this study showed similar changes in leaf and root growth rates, root cell viability, SOD, POD, and CAT activities, of K, Ca and Mg ion contents, protein contents, chlorophyll and MDA contents, and photosynthetic rates under AS and after RAS, but the magnitudes and response time of the changes differed depending on the maize line, suggesting differences in AS-tolerant mechanisms. The increased MAD contents in the tissues of the AS-treated maize lines (Fig. 3a, b) indirectly supported the previous conclusion that the Al treatment could trigger lipid peroxidation in the sensitive maize lines (Giannakoula et al. 2008), but they did not corroborate the view that Al treatments did not induce lipid peroxidation in both sensitive and tolerant maize lines (Boscolo et al. 2003). Our results showed that in maize, AS can cause decreases in the Ca and Mg ion contents in Al-tolerant Y478 and Z58 lines and Al-sensitive H4 and C7-2 lines (Fig. 5c–f), which supported previous conclusions (Giannakoula et al. 2008; Mihailovic et al. 2008). However, AS caused a significant decrease in K ion contents (Fig. 5a, b), which was in contrast to the previous conclusion (Yu et al. 2011). The discrepancies in the above-mentioned results likely resulted from the differences among the maize lines and/or partly from the experimental conditions, such as applied Al^3+^ concentrations and/or stress duration. During AS or after RAS, changes in the contents of Ca, Mg and K ions (Fig. 5) were closely related to changes in the cell structure in the root tip zone (Fig. 4), suggesting that low external Al concentrations can also lead to the loss of Ca, Mg, and K ions by disrupting the cell’s integrity. Additionally, the decreased ion contents in the roots of the AS-treated maize lines may be partially ascribed to impaired root uptake capacity during AS.

Considering the promotion of leaf growth (Fig. 1a–c) during AS as well as the recovery of AS-damaged cell walls in the root tip zone after RAS (Fig. 4) and other parameters of AS-treated maize lines after RAS treatment, we conclude that low doses of Al only decrease root growth rate and that the AS-caused inhibition of root growth of maize can be alleviated by appropriate RAS treatments.

For AS-treated maize lines, changes in the chlorophyll contents (Fig. 6c) did not correspond with changes in the photosynthetic rates (Fig. 6d), suggesting that the differences among photosynthetic rates in maize lines under AS result from differences in photosystems rather than chlorophyll contents. This reasoning partly confirms a previous finding that AS led to a severe decrease in activity of photosystem 2 activity (Mihailovic et al. 2008).

For an in-depth analysis of the correlation among the parameters, we conducted a multiple factor correlation analysis of the data resulting from AS and RAS treatments (Tables 1, 2).

**Table 1:** Correlation among affected parameters in the roots under AS and after RAS

Parameters	Al	Growth	SOD	POD	CAT	SOR	MDA	Protein	K	Ca	Mg
Al	1										
Growth	−0.873**	1									
SOD	−0.798*	0.664	1								
POD	−0.414	0.457	0.767*	1							
CAT	−0.808*	0.740*	0.725*	0.140	1						
SOR	0.711*	−0.711*	−0.754*	−0.090	−0.798*	1					
MDA	0.869**	−0.785*	−0.749*	−0.240	−0.747*	0.708*	1				
Protein	0.745*	−0.623	−0.453	−0.187	−0.453	0.730*	0.442	1			
K	−0.766*	0.639	0.610	0.372	0.716*	−0.276	−0.833*	−0.194	1		
Ca	−0.832*	0.849*	0.595	0.281	0.695	−0.672	−0.741*	−0.559	0.650	1	
Mg	−0.703*	0.463	0.553	0.453	0.594	−0.177	−0.605*	−0.127	0.849*	0.535	1

The analysis was conducted with all the data resulting from four the roots of Chinese maize foundation genotypes H4, C7-2, Y478 and Z58 under AS and after RAS according to Pearson correlation coefficients

* *P* < 0.05; ** *P* < 0.01

**Table 2:** Correlation among affected parameters in the leaves under AS and after RAS

Parameters	Al	Growth	SOD	POD	CAT	SOR	MDA	Protein	K	Ca	Mg	Chlorophyll	Photosynthetic rate
Al	1												
Growth	0.525*	1											
SOD	−0.596*	−0.415*	1										
POD	−0.552*	−0.214	0.483*	1									
CAT	−0.103	0.124	0.049	−0.569*	1								
SOR	0.578*	0.157	−0.262	−0.208	−0.077	1							
MDA	0.677*	0.169	−0.727**	−0.157	−0.450*	0.575*	1						
Protein	0.674*	0.418*	−0.677*	−0.258	−0.257	0.335	0.666*	1					
K	−0.791*	−0.347	0.406	0.111	0.456*	−0.606*	−0.728**	−0.714**	1				
Ca	−0.670*	−0.501*	0.225	0.244	0.017	−0.521*	−0.384	−0.624*	0.661*	1			
Mg	−0.568*	−0.202	0.535*	0.467*	0.040	−0.561*	−0.647*	−0.600*	0.529*	0.527*	1		
Chlorophyll	−0.506*	0.405*	−0.076	−0.051	0.508*	−0.128	−0.289	0.133	0.305	−0.268	0.055*	1	
Photosynthetic rate	−0.667*	−0.125	0.387	0.135	0.373	−0.667*	−0.731**	−0.345	0.699*	0.228	0.410	0.599*	1

The analysis was conducted with all the data resulting from four the leaves of Chinese maize foundation genotypes H4, C7-2, Y478 and Z58 under AS and after RAS according to Pearson correlation coefficients

* *P* < 0.05. ** *P* < 0.01

SODs together with PODs form the first line of antioxidant defense against ROS (Ito-kuwa et al. 1999; Veljovic-Jovanovic et al. 2006). In the SOD-POD system, SODs first degrade O_2_^−1^ into O_2_ and H_2_O_2_, and the latter is then degraded by POD into H_2_O and O_2_ (Boscolo et al. 2003; Wang et al. 2013). CAT scavenges photorespiratory H_2_O_2_ by a catalytic reaction of 2H_2_O_2_ → O_2_ + 2H_2_O (Willekens et al. 1997). As expected, there was a positive correlation between SOD and POD activities in the roots (Table 1) and leaves (Table 2) of AS-treated maize lines. Interestingly, the CAT activity showed a positive correlation with the SOD activity in the roots of the AS-treated maize lines (Table 1) but showed a negative correlation with the POD activity in the leaves of the AS-treated maize lines (Table 2). This suggests that the roots of the AS-treated maize lines require more antioxidant enzymes to cope with AS-triggered peroxidation relative to the AS-treated leaves. This appears reasonable because SOR production was greater in the AS-treated roots than in the AS-treated leaves (Fig. 3c, d). Thus, CAT is likely an auxiliary antioxidant enzyme that selectively cooperates with either SOD or POD to play a role in antioxidation under AS and after RAS, depending on maize tissues.

The CAT activity positively correlated with root growth rate (Table 1), while the SOD activity showed a positive correlation with the leaf growth rate (Table 2). This suggests that CAT is a major antioxidant enzyme responsible for root growth, and that SOD is an important enzyme for leaf growth under AS and after RAS.

There was a strong correlation between the Mg ion content and K ion content in the roots of the AS-treated maize lines (Table 1), and among Ca, K, and Mg ion contents in the leaves of the AS-treated maize lines (Table 2). This strongly suggests that there is a synergetic leakage from and/or uptake of Ca, K, and Mg ions by the roots under AS, depending on the maize tissues. The chlorophyll content positively correlated with the Mg ion content, but the photosynthetic rate positively correlated with the K ion content in the leaves of the AS-treated maize lines (Table 2), indicating differential differences in roles of Mg and K ions in photosynthesis under AS and during RAS.

The promotion of leaf growth in the AS-treated maize lines not only occurred during AS but also lasted during RAS (Fig. 1f), suggesting that the AS-promoting effect on leaf growth is in the ‘memory’ of AS-treated maize. Reportedly, the growth stimulation in plants receiving Al applications was ascribed not only to the alleviation of H^+^ toxicity but also to the increase in root uptake activity of nutrient elements, such as P (Osaki et al. 1997). However, this conclusion was not supported by the research in which the soybean roots were exposed to Al in a 0.5 mM Ca solution at pH 4.5 without other nutrients (Du et al. 2010). In the Al-accumulating plant *Mel.malabathricum*, Al, together with other nutrients, could promote the synthesis of adequate amounts of citrate (Watanabe et al. 2005) and could also induce a reduction in toxic Fe accumulation in roots and shoots (Watanabe et al. 2006). Therefore, the exact mechanisms for the AS-promoting effect on plant growth are still not fully understood (Ma 2007). The analyses indicated that AS-promoted leaf growth correlated positively to protein content and negatively to Ca ion content (Table 2). An increased protein content is undoubtedly conducive to plant growth at least because proteins are important “raw materials” (precursors) for important metabolites such as amino acids. Although the Ca ion content in roots and leaves of the AS-treated maize lines decreased (Fig. 5c, d), it correlated positively with root growth (Table 1) and negatively with AS-promoted leaf growth (Table 2). These results supported the conclusion that elevating Ca inhibits shoot growth and promotes root growth (Hepler 2005). This may be because reducing the Ca ion concentration promotes cell and tissue elongation and elevating Ca ion inhibits cytoplasmic streaming (Hepler 2005). The stresses, to some extent, lead to ubiquitin-mediated proteasomal degradation of growth-repressing proteins, such as DELLA in plants and consequently promote growth (Conti et al. 2014). Therefore, another reason for AS-promoted leaf growth is also likely associated with the discharge of growth-inhibitory factors from the growth-regulating molecules under AS.

## Conclusion

Low doses of Al inhibit root growth but enhance leaf growth in maize lines. The AS-promoted leaf growth is likely associated with increased protein synthesis, a lowered Ca ion content, and the discharge of growth-inhibitory factors from the growth-regulating molecules. Some unknown compensating mechanisms regulate AS-promoted leaf growth. Additionally, AS-promoted leaf growth is in the ‘memory’ of AS-treated maize plants. CAT is an auxiliary antioxidant enzyme that work selectively with either SOD or POD against AS-caused peroxidation. CAT is a major antioxidant enzyme responsible for root growth, but SOD is important for leaf growth under AS and during RAS.

### *Author contribution statement*

L.W. conducted all experiments; X.-W.F. assisted in the design of some of the experiment programs and participated in discussion of the results; J.-L.P. and Z.-B.H. helped L.W. perform parts of the experiments; Y.-Z.L. was in charge of the research project and finished the manuscript.

## Abbreviations

AS: Al stress
CAT: Catalase
MDA: Malondialdehyde
POD: Peroxidase
RAS: Removal of AS
ROS: Reactive oxygen species
SOD: Superoxide dismutase
SOR: Superoxide radicals
